# Tenth International Symposium on the Hsp90 chaperone machine: Switzerland, October 19-23, 2022

**DOI:** 10.1007/s12192-023-01342-z

**Published:** 2023-04-18

**Authors:** Adrienne L. Edkins, Markus Zweckstetter, Ritwick Sawarkar

**Affiliations:** 1 Biomedical Biotechnology Research Unit (BioBRU), Department of Microbiology and Biochemistry, Rhodes University, Makhanda, South Africa; 2 German Center for Neurodegenerative Diseases (DZNE), Göttingen, Germany; 3 Department for NMR-Based Structural Biology, Max Planck Institute for Multidisciplinary Sciences, Göttingen, Germany; 4 Medical Research Council (MRC) and Department of Genetics, University of Cambridge, Cambridge, UK

**Keywords:** Hsp90, Molecular chaperone, Cochaperones and client proteins

## Abstract

Hsp90 is a molecular chaperone responsible for regulating proteostasis under physiological and pathological conditions. Its central role in a range of diseases and potential as a drug target has focused efforts to understand its mechanisms and biological functions and to identify modulators that may form the basis for therapies. The 10^th^ international conference on the Hsp90 chaperone machine was held in Switzerland in October 2022. The meeting was organized by Didier Picard (Geneva, Switzerland) and Johannes Buchner (Garching, Germany) with an advisory committee of Olivier Genest, Mehdi Mollapour, Ritwick Sawarkar, and Patricija van Oosten-Hawle. This was a much anticipated first in-person meeting of the Hsp90 community since 2018 after the COVID-19 pandemic led to the postponement of the 2020 meeting. The conference remained true to the tradition of sharing novel data ahead of publication, providing unparalleled depth of insight for both experts and newcomers to the field.

## Introduction

**Fig. 1:**
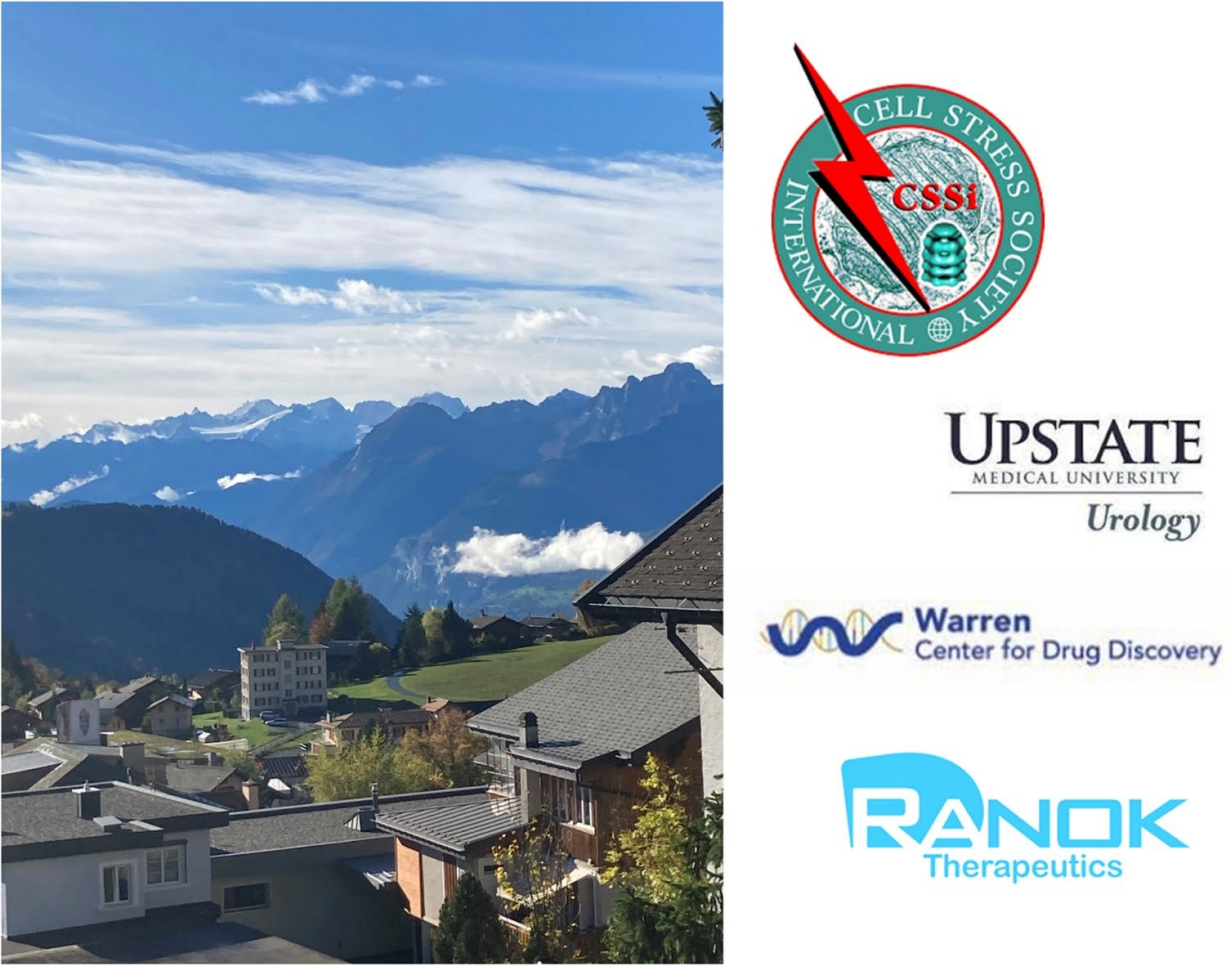
The view of Leysin from the Alpine Classic Hotel and our generous sponsors. Photograph taken by Adrienne Edkins

**Fig. 2:**
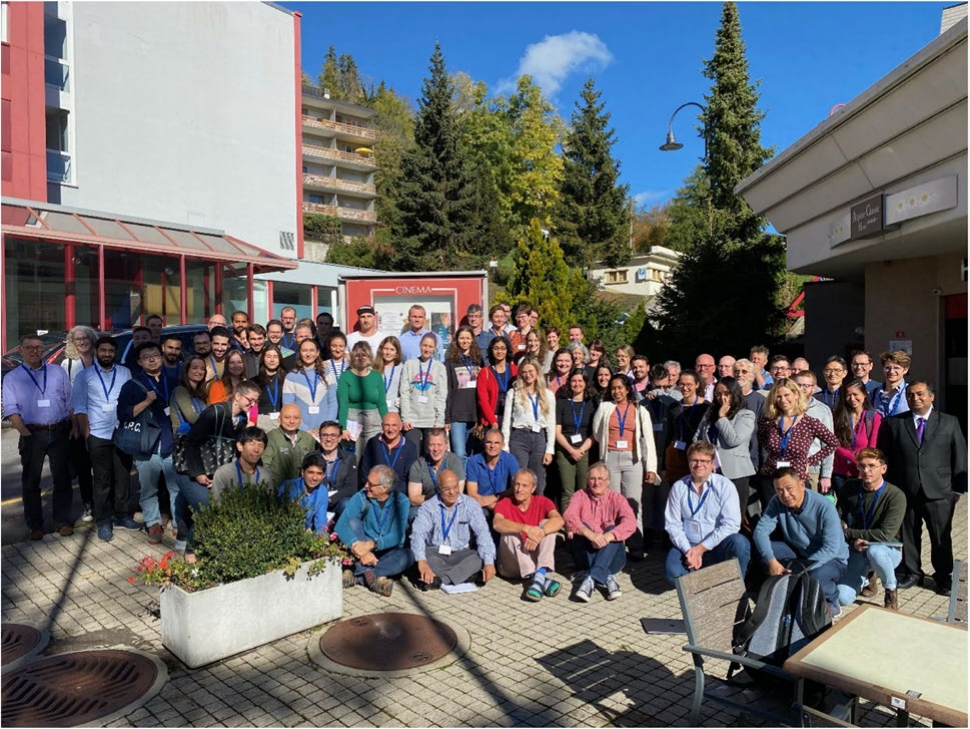
The group photo of the participants of the Hsp90 chaperone machine 2022. Photograph taken by Samarpan Maiti

The beautiful Swiss Alpine town of Leysin was the venue for the 10^th^ international conference on the Hsp90 chaperone machine in October 2022 (Fig. 1). After the 2020 meeting was postponed due to the COVID-19 pandemic, this was the first and much anticipated in-person meeting on the Hsp90 chaperone machine since 2018. With representatives from four different continents, there were 97 participants, 15 invited speakers, 25 short talks, and 67 abstracts received (Fig. 2). The conference lived up to expectations, providing stimulating engagement around several aspects of Hsp90 function, from structural and biophysical analysis of the chaperone machine to interaction with clients and cochaperones, posttranslational modifications, and the function of organelle-specific Hsp90 isoforms. The meeting was generously supported by the Cell Stress Society International, Ranok Therapeutics, Warren Center for Drug Discovery, and Upstate Medical University Urology.

## Hsp90 structure–function relationships

The Hsp90 chaperone complex undergoes a series of conformational changes in response to ATP binding and hydrolysis or cochaperone binding, and these are essential for its chaperone function. Keeping with tradition, the conference had a strong focus on understanding structure–function relationships and describing the molecular mechanisms of the Hsp90 chaperone machine.

The conference started with a keynote lecture by Prof. Sebastian Hiller (Biozentrum, University of Basel). He presented high-resolution studies of the functional cycle of the Hsp70 chaperone BiP that plays a central role in the dynamic chaperone network of the endoplasmic reticulum (ER). Using solution NMR spectroscopy, he showed that it is possible to resolve individual states of this protein at atomic resolution and with high temporal resolution during its functional cycle. Further studies included a disulfide isomerase that plays a crucial role in the ER and appears to respond to ER stress in a noncanonical way.

Sonja Schmid (Wageningen University, Netherlands) presented work studying Hsp90 with single-molecule resolution. She introduced the concept of Hsp90 stimulation through conformational confinement, i.e., by reducing floppy off-states of Hsp90, which is possible even nonspecifically using macromolecular crowding (Schmid and Hugel 2020). Using single-molecule Förster resonance energy transfer (smFRET (Hellenkamp et al. 2018; Gotz et al. 2022)) studies she showed that various stimuli, including point mutations, cochaperones, and macromolecular crowding, affect Hsp90’s conformational equilibrium in a similar way. However, the underlying kinetic rates are different (Schmid and Hugel 2020), demonstrating the added functional insight gained from single-molecule kinetics data. In addition, she harnesses new label-free nanopore techniques (Schmid and Dekker 2021a) to study Hsp90, for example using their recently developed NEOtrap (Schmid et al. 2021), to tackle persistent questions relating to the Hsp90 mechanism with new time-resolved single-molecule techniques (Schmid and Dekker 2021b).

Daniel Bolon (University of Massachusetts Chan Medical School, USA) presented deep mutational scanning analyses of Hsp90 aimed at identifying critical properties that underlie function. Results from his lab showed that proximity to the gamma-phosphate of ATP was sufficient to explain most of the mutational sensitivity for yeast growth under standard conditions (Flynn et al. 2020; Mishra et al. 2016). The lab is currently exploring the Hsp90 mechanism by investigating dominant mutations. Jill Johnson (University of Idaho, USA) presented data on a new series of yeast Hsp90 mutants that disrupt distinct conformations within the folding cycle. The Johnson group used those mutants to gain new insights into how cochaperones regulate the cycle, finding that Hch1 has a unique role as a negative regulator of progression to the closed conformation. In contrast, Cpr6 promotes progression to the closed conformation, but it appears as though Cpr6 release is necessary for steps involved in reopening. Michael Reidy from the laboratory of Daniel Masison (National Institutes of Health/NIH, USA) reported unpublished findings regarding the importance of ATP hydrolysis in Hsp90’s in vivo functions.

## Hsp90 interactions with cochaperones, chaperones, and client proteins

Hsp90 does not work in isolation but forms functional complexes that include client proteins, cochaperones, and other chaperones. Pierre Goloubinoff (Tel Aviv University, Israel) discussed that chaperones, such as Hsp60, Hsp100, and Hsp70, are enzymes that use energy from ATP hydrolysis to unfold and thus repair structurally damaged polypeptides. His lab showed that DnaJ targets Hsp70 exclusively unto aggregates and avoids the native conformation (Rebeaud et al. 2021). In addition, evolutionary studies of the main chaperone families (Tiwari et al. 2023) showed that simple free-living bacteria can live without Hsp70, Hsp90, and Hsp100. He concluded that Hsp70 acts as an obligate cochaperone of Hsp90 in eukaryotes and prokaryotes, containing the Hsp90 ortholog HTPG, show that Hsp70 without Hsp90 has a stand-alone unfolding/disaggregation activity, but Hsp90 without Hsp70 lacks significant chaperone activity. Anushka Wickramaratne from Sue Wickner’s group (NCI/NIH USA) presented her findings that *E. coli* Hsp90 (HTPG) forms a ternary complex with CbpA and DnaK (bacterial Hsp70). This complex is anticipated to play a role in client remodeling.

Gabriela Chiosis (Memorial Sloan Kettering Cancer Center, USA) emphasized the central role of higher order heterooligomeric complexes of Hsp90, cochaperones, clients, and other factors to the development of human disease (Ginsberg et al. 2023; Joshi et al. 2021). Brian Freeman (University of Illinois, USA) presented data on a global analysis of the physical Hsp90 interactome and provided insight into client selection by Hsp90, the domains used by Hsp90 for interaction with client proteins and novel biological pathways involving Hsp90 (Mankovich and Freeman 2022). David Agard (University of California, San Francisco, USA) and Chari Noddings from the Agard group described the atomic structures of chaperone complexes which provide mechanistic insight into the stages of client folding by the chaperone machine. Using cryo-EM structures of chaperone complexes and glucocorticoid receptor (GR) as a model client, they described the mechanisms of a complete chaperone cycle involving Hsp90, Hsp70, and selected cochaperones (Noddings et al. 2023; Noddings et al. 2022; Wang et al. 2022). Ineke Braakman (Utrecht University, Netherlands) discussed the role of the Hsp90 complex as a triage system for mutant CFTR function, showing that specific domain mutants give rise to the most profound folding defects which influences chaperone interactions (Im et al. 2023).

Asat Baischew from Felix Hausch’s group (TU Darmstadt, Germany) presented the site-specific incorporation of photoreactive unnatural amino acids in a high-throughput manner to map the in-cell interaction of the Hsp90 client GR with the large immunophilins FKBP51 and FKBP52. According to Asat Baischew, the data suggest the orientation of the apo-GR towards the immunophilins and support a large and clearly defined interaction interface. Selected mutants were used as proximity sensors to characterize the in-cell interaction with chemical tools.

Chrisostomos Prodromou (University of Sussex, UK) presented data showing that a small fragment of the N-and the C-terminal domain of the Hsp90 cochaperone CDC37 was sufficient for binding to the BRAF kinase. This bipartite interaction requires a minimum spatial separation for high affinity binding, and a specific motif, located in the N-terminal domain of CDC37, recognizing the C-lobe of the BRAF kinase-domain was presented. In addition, the class 2 L597R mutant of BRAF was shown to be a homodimer but unexpectedly had no kinase activity. It would appear that its signaling would be dependent on a RAS-driven heterodimerization with CRAF, rather than independently by BRAF homodimers. The potential clinical significance of this is that cancers driven by L597R should be targeted with CRAF-specific inhibitors rather than those for BRAF.

Jasmeen Oberoi from the Laurence Pearl group (University of Sussex, UK) presented the cryoEM structure of an Hsp90-CDC37-BRAF^V600E^ complex, which revealed how the oncogenic BRAF^V600E^ kinase is primed for association with Hsp90-CDC37. She also showed structures of Hsp90-CDC37-BRAF^V600E^ complexes bound to PP5, in both an autoinhibited and activated conformation, together with subsequent proteomic analysis, which identified sites of PP5 dephosphorylation within the BRAF^V600E^ and CRAF kinases. These findings reveal how PP5 is activated by recruitment to Hsp90 complexes to comprehensively dephosphorylate Hsp90-dependant client kinases, consequently disrupting interactions with regulatory proteins such as 14–3-3 and performing a “factory reset” of the kinase prior to release.

Mehdi Mollapour (State University of New York (SUNY) Upstate Medical University, USA) demonstrated that the cochaperones FNIP1, FNIP2, and Tsc1 form distinct heterocomplexes that regulate Hsp90 chaperone activity and differentially activate kinase and nonkinase client proteins, including steroid hormone receptors. Furthermore, FNIP1, FNIP2, and Tsc1 alone and in combination enhance steroid hormone receptor ligand binding and activity. He proposed a model where the formation of different populations of Hsp90: cochaperone: client complexes provides differentially primed pools of client proteins ready to act in different cellular environments (Backe et al. 2022).

Florent Delhommel from Michael Sattler’s group (Technische Universität München, Germany) described a collaboration between the labs of Johannes Buchner, Michael Sattler, and Rina Rosenzweig on the structural and functional characterization of the essential cochaperone NudC (Biebl et al. 2022), initially identified through a CRISPR interference assay on proteostasis-related genes. He proposed that the combination of NMR, crystallography, and modeling shows how NudC binds to Hsp40, Hsp90, and the client GR. NudC mediates the transfer of clients to Hsp90 by recruiting Hsp40-bound substrates, while excluding Hsp70, thus accelerating client activation. NudC shares a CS domain with the cochaperone p23, which has been previously shown to modulate dynamic Hsp90 conformations with client binding (Biebl et al. 2021; Lopez et al. 2021).

Adrienne Edkins (Rhodes University, South Africa) described the identification of a novel isoform of the Hop gene which produces a protein variant with altered subcellular localization and Hsp90 interaction. Hop is an early-stage cochaperone of the Hsp90 complex involved in regulating client entry (Schwarz et al. 2023). This Hop isoform is believed to expand the complexity of Hop-mediated protein transfer and may represent a mechanism by which to recruit Hsp90 to distinct locations within the cell under specific conditions.

Sonja Engler from Johannes Buchner’s lab (Technische Universität München, Germany) discussed studies investigating the currently largely unknown role of the cochaperone Sgt1 in the Hsp90 chaperone cycle. In these studies, they discovered that maturation of GR in vivo and in vitro is Sgt1-dependent. Further structural and biochemical analyses showed that Sgt1 is involved in the loading of clients onto Hsp90.

R2TP is a highly conserved Hsp90-containing chaperone complex formed by two AAA + ATPases, RUVBL1 and RUVBL2, that associate with PIH1D1 and RPAP3 proteins. R2TP functions with several other chaperones including Hsp90 and Hsp70 to promote macromolecular complex assembly. Walid Houry (University of Toronto, Canada) reported the identification of a novel protein, termed DPCD, as a new component of R2TP that allows R2TP to modulate the formation of cilia. These findings highlight a new function of R2TP in maintaining cellular protein homeostasis.

## Hsp90 posttranslational modifications

Hsp90 is also subject to regulation by posttranslational modifications (PTMs) including phosphorylation. This layer of posttranslational regulation has been termed the “chaperone code” and is providing a new level of complexity to the mechanisms used to fine-tune Hsp90 function. Matthias Mayer and his group at the Center for Molecular Biology of Heidelberg University (ZMBH), Germany, reported on studies investigating the influence of tyrosine phosphorylation of human Ηsp90α on client maturation. Expressing phosphomimetic glutamate and nonphosphorylatable phenylalanine variants of human Hsp90α in a yeast model system, the Mayer group found that different clients, even closely related steroid hormone receptors, have markedly different preferences for the different Hsp90 variants. The same variants stabilized distinct clients to different degrees on transient transfection in HSP90AA1-deleted HepG2 cancer cells. In vitro, these variants had distinct interaction patterns with cochaperones p23, Cdc37, Aha1, and Hop. Matthias Mayer proposes that posttranslational modifications constitute a code to adapt Hsp90s for optimal chaperoning of the dominant clients of a cell.

Many PTMs have been reported for Hsp90, but the functional impact has been characterized for only a few of them. Manfredo Quadroni (University of Lausanne, Switzerland) discussed an investigation into the phosphorylation of Ser 226 and Ser 255 in human Hsp90β. Surprisingly these PTMs, located in the poorly conserved and yet functionally important charged linker (CL), appeared to be present at invariantly high stoichiometry (> 95%) under most conditions tested. A nonphosphorylatable Ala/Ala mutant reproducibly bound a higher amount of the most known interactors and was more sensitive to limited proteolysis, suggesting a more open conformation. Perhaps more importantly, the nonphosphorylated AA mutant appeared to be secreted more efficiently, which suggests that phosphorylation in the CL could be an intracellular retention signal.

Dr. Sarah Backe from Dimitra Bourboulia’s lab (SUNY Upstate Medical University, USA) presented recent work that also linked PTM of components of the Hsp90 chaperone complex in the extracellular environment. She showed that the protooncogene c-Src tyrosine kinase stimulates enrichment of extracellular phosphotyrosine-containing proteins including components of the Hsp90 chaperone machinery. c-Src kinase phosphorylates the extracellular Hsp90 (eHsp90) cochaperone TIMP2, which impacts directly on eHsp90 client MMP2 proteolytic function (Sanchez-Pozo et al. 2018). Considering that c-Src levels and activity are elevated in many human cancers, enhancement of tyrosine phosphorylation of the cancer phosphoproteome would have an impact on cell–matrix and cell–cell communication. Indeed, gene ontology analyses revealed enrichment of cancer promoting pathways, largely because of the phosphorylation of chaperone proteins. It was proposed that these findings may have a broader impact on extracellular kinase/chaperone signaling and emphasize the prospect of utilizing this information in cancer diagnosis and the design of new anticancer therapies.

## Organelle-specific Hsp90 isoforms

Besides the cytosol and the nucleus, Hsp90 plays critical roles in the physiology of organelles such as mitochondria, ER, and the chloroplast. Several speakers presented unpublished data on organellar Hsp90 and its relevance in diseases. Dan Gewirth (Hauptman-Woodward Institute, USA) presented work highlighting the role of glycosylation at the five minor sites in regulating the function of Grp94, the ER Hsp90 paralog. Using singly modified proteins, his group showed that glycosylation at some of the minor sites leads to instability, aggregation, inability to bind cochaperones, premature cellular clearance, and loss of the Grp94 client maturation function. In contrast, modification at nondeleterious sites led to insights into how differential glycosylation can tune Grp94 chaperone activity and promote new biological functions.

TRAP1 is a mitochondrial Hsp90 that is a master regulator of global metabolic flux. Marc Woodford (SUNY Upstate Medical University, USA) described work to determine the impact of the glucose-derived modification O-GlcNAcylation on TRAP1 function. He showed that TRAP1 O-GlcNAcylation occurred within mitochondria and suppressed TRAP1 ATPase activity. TRAP1 loss decreased cellular response to inhibition of the O-GlcNAcase enzyme, suggesting a reciprocal regulatory mechanism for TRAP1-GlcNAc that impacts global cell metabolism.

Byoung Heon Kang (Ulsan National Institute of Science and Technology, South Korea) gave an overview of the current status of orthosteric and allosteric TRAP1 inhibitors and described their mode of target binding at the molecular level. Mitochondria-targeted antioxidant mitoquinone (MitoQ) was identified as an allosteric TRAP1 inhibitor targeting the client binding site, and the structural and biochemical analyses provided molecular details on the drug binding pocket in the middle domain of TRAP1. This pocket is conserved in other Hsp90 family proteins and may also be targeted to develop allosteric inhibitors. TRAP1 knockout dramatically reduced retinal pathogenesis in mouse retinopathy models in vivo, and TRAP1 inhibitors reduced aberrant vascular changes in the retinopathy mice, identifying TRAP1 inhibitors as a potent treatment for ischemic retinal diseases.

Work presented by Timothy Street (Brandeis University, USA) focused on three findings about the Hsp70/Hsp90 system in the endoplasmic reticulum (BiP/Grp94). He demonstrated that BiP acts as a closure-accelerating Grp94 cochaperone and that electrostatic steering drives BiP to specifically bind oligomerized states of the client protein proIGF2. This work showed that single-molecule FRET is a promising method for uncovering how BiP controls the position of clients on Grp94.

Rongmin Zhao (University of Toronto, Canada) presented his research focusing on the plant Arabidopsis ER GRP94 orthologue which they referred to as Hsp90.7. He discussed that this plant GRP94 protein contains a unique feature in the middle domain and how this protein could play a positive role specifically under ER stress conditions. Zhao also reported their work on a new mutant line that exemplifies the essential role of the protein in regulating tissue and organ differentiation at both shoot and root apical meristems.

Hsp90C, the chloroplast homologue of Hsp90, is involved in protein import and is believed to transport essential actors of photosynthesis to the thylakoids. Romain La Rocca from Philippe Meyer’s lab (CNRS UMR 8226, Paris, France) showed that *Arabidopsis thaliana* Hsp90C showed remarkable differences compared to other Hsp90 family members. Firstly, Hsp90C had a 10-to-100-fold higher ATPase activity partly stimulated by an atypical N-terminal extension. Secondly, its crystal structure showed a rearranged homodimer with wider lumen suggesting it could interact with its clients through a noncanonical mechanism.

## Physiological and therapeutic modulation of Hsp90

Yuantao Huo from Matthias Mayer’s group (ZMBH, Heidelberg University) established a doxycycline-induced transcriptional silencing system to achieve the knockdown of Hsp90α and Hsp90β, alone, and in combination and explored the impact on cells when Hsp90 is absent. This reversible knockdown system could effectively remove most of the target protein in a controllable manner. Interestingly, there was no significant change in cells upon the single isoform knockdown, but when the double knockdown was employed, cells suffered from the loss of both Hsp90s. A strong compensation was observed between the two isoforms, and heat-shock responses were also activated to rescue the cells from loss of Hsp90. Brian Blagg (University of Notre Dame, USA) discussed selective chemical inhibition of specific Hsp90 isoforms. Despite high levels of structural similarity between selected domains in Hsp90 isoforms, inhibition of specific isoforms can be achieved, and this gives rise to distinct phenotypes compared to pan-Hsp90 inhibition (Mishra et al. 2021). An alternative strategy for therapeutic targeting of Hsp90 complexes was presented by Kevin Foley (Ranok Therapeutics, USA/China). He discussed the innovative platform developed by Ranok Therapeutics to use chaperone-mediated protein degradation (CHAMP™) for targeted Hsp90-mediated degradation of disease relevant proteins.

Samarpan Maiti discussed his work in the Picard laboratory (University of Geneva, Switzerland) on the link between cell size and stress adaption. He demonstrated that mammalian cells gradually enlarge their size to adapt to mild but chronic stress in an HSF1-dependent fashion and that their adaptation to chronic stress is different from the response to acute stress. Unlike acute stress, which causes a shutdown of global translation to reduce the protein burden, chronic stress induces global translation to increase the total amount of protein. Hsp90, irrespective of the isoform expressed, supports the increase in global translation.

Given its specific biochemical properties, it is not surprising that Hsp90 is at the center of several cellular functions associated with protein folding. Misfolding and aggregation of the microtubule-associated protein Tau inside neurons are pathological hallmarks of Alzheimer’s disease. Markus Zweckstetter (German Center for Neurodegenerative Diseases, and Max Planck Institute for Multidisciplinary Science, Göttingen, Germany) presented recent data from his lab in which they showed that phosphorylated Tau binds with high affinity to the Hsp70/Hsp90-chaperone machine. The complex of Tau with the high-molecular weight complex formed by Hsp70, Hop, and Hsp90 is stabilized by binding of the Hsp90 cochaperone p23 and can be efficiently disintegrated by the E3-ubiquitin ligase CHIP, which targets Tau to proteasomal degradation.

Paul LaPointe (University of Alberta, Canada) presented his work on how inhibition of Hsp90 results in changes in the immunopeptidome of cancer cells. These changes correlated with enhanced recognition of cancer cells by T-cells suggesting that disruptions in proteostasis may augment immune checkpoint blockade therapy.

## Organismal functions of Hsp90

The function of Hsp90 at the organismal level has been intensely investigated using model organisms. A report that Hsp90 inhibition reveals heritable eye-size variation in *Astyanax mexicanus* cavefish bolstered the Hsp90 “evolutionary capacitor hypothesis,” which theorizes that Hsp90 may provide molecular buffering by stabilizing mutant proteins (Rohner et al. 2013). Cavefish provide a unique model system in which to explore this hypothesis as their evolutionary history includes a specific environmental induction of heat shock response (low conductivity water in the caves) and Hsp90 inhibition reveals phenotypic variants in a specific selected trait (eye size/loss). The work presented by Hannah Grunwald from Clifford Tabin’s group (Harvard Medical School, Boston, USA) aimed at identifying the Hsp90-dependent proteins responsible for eye size variation. By treating cavefish with inhibitors of specific cochaperones (e.g., quercetin to test the necessity of Cdc37-Hsp90 in buffering phenotypic variation), and using existing quantitative trait loci datasets, the work is geared to identify candidate genes that are both Hsp90 clients and also linked to loci that control eye size in the cavefish. Using CRISPR knock-outs in zebrafish, they hope to identify novel genes that control eye size in an Hsp90-dependent manner.

Patricija van Oosten-Hawle (University of North Carolina at Charlotte, USA) presented data showing how Hsp90 knockdown in the *C. elegans* gut initiates a “gut-to-muscle” stress signaling route that enhances lifespan and organismal proteostasis. Her group identified key signaling components that relay the stress signal from the gut to the muscle, involving secreted lipases and other signaling components leading to the activation of *hsp-70* in a manner antagonistic to HSF-1.

Sunanda Bhattacharyya (University of Hyderabad, India) described the regulatory function of Hsp90 during homologous recombination mediated DNA double strand break repair. She showed that in response to DNA damage, yeast Hsp82 is translocated to the nucleus from cytoplasm in an Aha1-dependent manner and recruited to the broken DNA ends until the repair.

## Contribution to the field: Didier Picard

In addition to the excellent scientific program, the 10^th^ international conference on the Hsp90 chaperone machine marked another milestone: the retirement of Didier Picard (Geneva, Switzerland). Didier, together with Johannes Buchner, has been a champion of the Hsp90 community for many years and was the driving force behind establishing the meeting in 2002. Johannes paid tribute to the different ways in which Didier has contributed to the field, noting how Hsp90 has not only “shaped proteins” but has also “shaped lives.” Didier has been at the forefront of Hsp90 research since his initial descriptions of the link between Hsp90 and steroid hormone receptors (Picard et al. 1990), he established many core research tools used by the field, and his laboratory website is a legendary and accessible curated resource on Hsp90-related literature (https://www.picard.ch and https://www.hsp90.org/). In establishing the Hsp90 conference, he was part of creating a collaborative, welcoming international community of Hsp90 researchers. When the COVID-19 pandemic prevented in-person meetings, Didier responded by initiating the online Hsp90 webinar series to ensure continuity in the community. We cannot underestimate the importance and value Didier has brought to our field over the years. This meeting, 20 years after the first, was a fitting time and place to reflect and acknowledge the impact Didier has had on the field. And thankfully, Didier will return as an “honorary organizer” for the 11^th^ international conference on the Hsp90 chaperone machine which will be held between the 23rd and 27th of October 2024 in Seeon, Germany. No doubt this meeting will continue the strong tradition set by Didier and Johannes over the years, and we look forward to seeing you there!

## Data Availability

All data are provided in the manuscript.
